# Off-label use of teriparatide for the treatment of a vertebral burst fracture in a young patient: A case report and literature review^☆^

**DOI:** 10.1016/j.tcr.2025.101127

**Published:** 2025-01-04

**Authors:** Tiziano Villa, Vincenzo Zottola, Carlo Mariani, Alberto Borgonovo, Luciano Redenti

**Affiliations:** a G. B. Mangioni Hospital GVM, Lecco, Italy; b Fondazione IRCCS San Gerardo dei Tintori, Monza, Italy; c Salus Hospital GVM, Reggio Emilia, Italy; d Studio medico-legale Redenti, Como, Italy

**Keywords:** Teriparatide, Electro-magnetic field, Conservative treatment, Thoracolumbar burst fracture, Young patient

## Abstract

We describe a case of L2 vertebral burst fracture in a 40-year-old male patient without any other comorbidities due to a ground-fall from horse. Despite surgical intervention with L1-L3 spine fusion was indicated as the treatment of choice since no fracture healing was visible on CT after 40 days post trauma, a conservative off-label therapy was decided with 5-month administration of teriparatide and vitamin D‑calcium supplementation together with nocturnal pulsed electro-magnetic field. Complete fracture healing was obtained after 5 months.

This is the first report of successful off-label use of teriparatide together with pulsed electro-magnetic field for the treatment of a burst vertebral fracture without surgical intervention in a young patient.

## Introduction

Among 90 % of all spinal fractures comprise thoracolumbar fractures, while nearly 14 % of all spinal fractures are accounted as burst fractures [1]. These ones are considered as compression fractures secondary to high-energy trauma, such as falls from height, sports and traffic injuries. The thoracolumbar segment (T10-L2) represents the region most commonly involved with traumatic spinal fractures and the most exposed to neurological injury risk since, at the level of L1-L2, the spinal cord ends, with the roots of the cauda equina filling the canal [2].

Treatment of thoracolumbar vertebral fractures can be both surgical and non-surgical. Surgical treatment may allow immediate mobilization and earlier rehabilitation. Indications to surgery involve mainly neurological deficit, and type B and C fractures, kyphotic deformity of >15–20°, scoliotic deformity >10° or relevant traumatic disc damage [3]. On the other hand, indications to conservative treatment are type A stable compression and burst fractures with canal encroachment in the absence of a neurologic function's major deficit [4].

Conservative treatments usually require initial rest in bed, followed by an extension cast or brace and early mobilization, which is important for a better clinical outcome [5]. Moreover, the involvement of drug use in the conservative treatment allows to give a valuable alternative to the surgical treatment. In this respect, a novel drug is Teriparatide (Forsteo®, Eli Lilly & Co Ltd., Liverpool, England), a recombinant human parathyroid hormone (PTH 1–34), which promotes osteogenic activity, and therefore bone healing. The drug is approved for bone fragility fractures in patients already treated with bisphosphonates.

Some studies have shown teriparatide to be effective, as well as internal surgical fixation, for the management of bone fractures, but there's poor evidence of its use, combined with Pulsed Electro-Magnetic Field (PEMF), for the treatment and prognosis of vertebral burst fractures in young patients. We report herein the effectiveness of the combined treatment of teriparatide with PEMF for a stable lumbar vertebral fracture in a young patient.

## Case description

We present the case of a 40-year-old male patient who sustained a L2 vertebral burst compression fracture with a large frontal bone fragment, after falling from a horse, treated with off-label use of teriparatide.

**Fig. 1:**
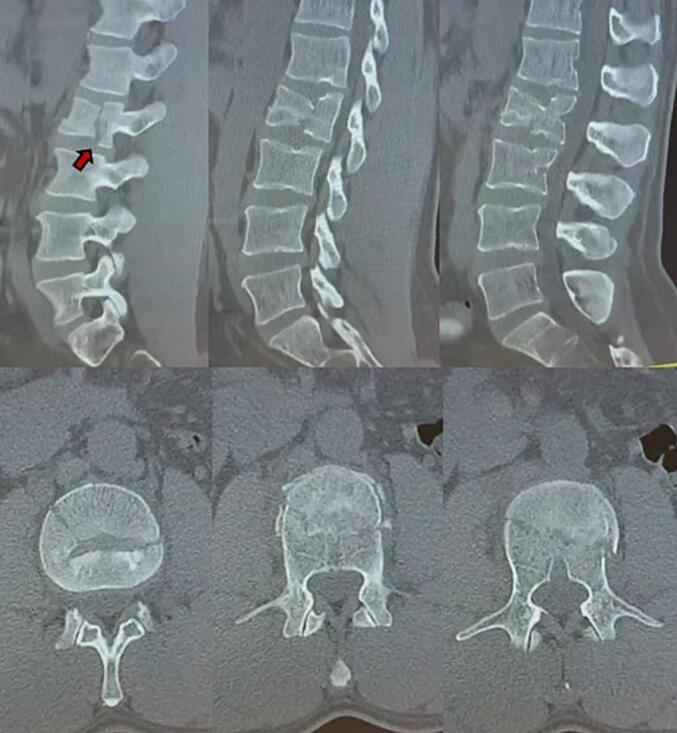
Computed Tomography (CT) scan of vertebral column, immediately after the trauma (sagittal and transverse views). Red arrow highlights the L2 vertebral compression fracture, with voluminous anterior bone fragment. (For interpretation of the references to colour in this figure legend, the reader is referred to the web version of this article.)

At initial visit, the vertebral fracture has been classified as A2/A3 type according to the AO spine thoracolumbar classifications system through CT/MRI, without involvement of the spinal cord canal or damages to other soft tissues. The fracture was stable without any compromission of the posterior vertebral wall (Fig. 1). Surgical indication was immediately given due to the presence of a voluminous anterior bone fragment, comminution and risk of anterior wall separation.

Initially, it was nevertheless decided for conservative treatment with rest, waiting and seeing if the fracture would have spontaneously healed since the young age of the patient. The patient started to use a C35 rigid back brace by night and day for the first 2 months and only during day for the third month, in order to keep the fracture stable, avoiding any other damages. The patient continued to work, without driving for 35 days.

To reduce back pain, morphine (5 mg twice per day) was subcutaneously administered for 2 days, paracetamol (1000 mg twice per day) for 15 days and dexibuprofene (400 mg twice per day) for 6 days.

**Fig. 2:**
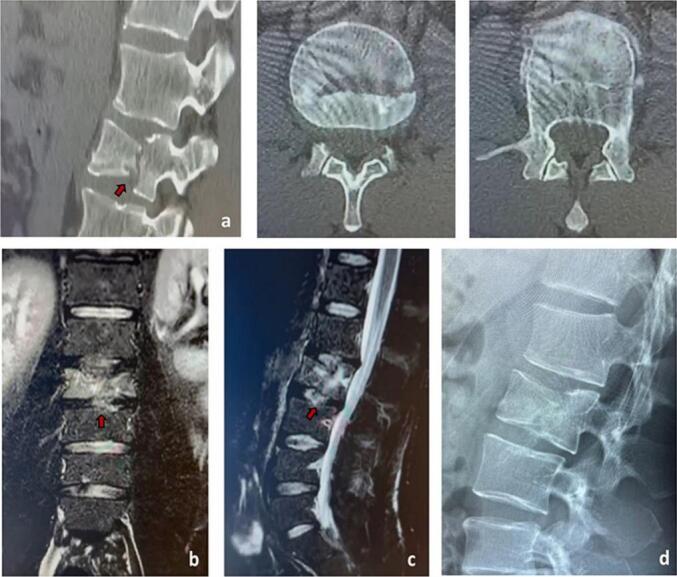
a) Computed Tomography (CT) scan at 40 days after the trauma (sagittal and transverse views), further showing L2 vertebral compression fracture with no improvement and no bone healing (red arrow). b-c) Magnetic Resonance Imaging (MRI) at 40 days after the trauma (coronal and sagittal views) showing comminuted L2 vertebral fracture and bone edema around the fracture. d) Radiography at 40 days after the trauma (sagittal view), showing L2 vertebral fracture. (For interpretation of the references to colour in this figure legend, the reader is referred to the web version of this article.)

However, after 40 days from the start of conservative treatment, there was no improvement in bone healing process, but an extensive bone edema was present (Fig. 2). Thus, the previous recommendation of surgical treatment with spine fusion and later implant removal was strengthened.

To avoid surgery and its related possible complications, the patient did not give his consent to undergo surgery and, in agreement with his spine surgeon and neurologist, decided to undergo an off-label treatment combining nocturnal Pulsed Electro-Magnetic Field (PEMF) therapy for 6–7 h/day with daily subcutaneous injection of 20 μg of Teriparatide (Forsteo®, Eli Lilly & Co Ltd., Liverpool, England), and with further administration of calcium (2 tablets/day of cal-mag-vitD3) and vitamin D (14 drops of Didrogyl® once a week) supplements for the subsequent 5 months, to enhance bone healing.

In addition, a cane has been used as a support and to prevent the occurrence of other fractures and/or injuries to the spinal cord. Moreover, physiotherapy was performed with 10 min of elliptical machine twice a day, and 10 min of rowing ergometer, for the first 15 days after rigid back brace removal. The patient underwent periodic radiological follow-up.

**Fig. 3:**
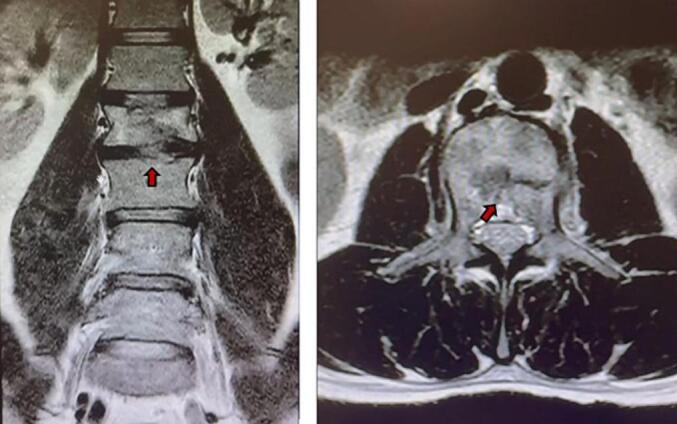
Magnetic resonance imaging (MRI) at final follow-up (5 months and 40 days after the trauma) demonstrating fracture healing (coronal and transverse views).

After this period of 5 months, complete healing of the vertebral fracture was shown by Magnetic Resonance Imaging (MRI) (Fig. 3).

Laboratory exams were performed to inspect the degree of inflammation, and they showed optimal results, with testosterone and thyroid levels within normal parameters.

After complete fracture recovery, the patient does not complaint pain anymore but only a moderate stiffness of the spine segment involved. Nevertheless, he was able to move as before the trauma and to return back to horse riding.

## Discussion

A novel conservative management for bone fractures is Teriparatide, which is the N-terminal aminoacidic portion of the human parathyroid hormone (PTH 1–34) [6]. Teriparatide is used in adults with osteoporosis, and it's subcutaneously injected with a recommended dose of 20 μg/day for a period of at least 6–12 months and for a maximum of 24 months [7].

Besides Teriparatide, another non-surgical treatment is represented by bisphosphonates, which are the most popularly prescribed anti-osteoporotic agents. They can increase the bone mineral density (BMD) by 4–8 %, by inhibiting bone resorption [6]. However, these patients have lost >25 % of their skeletal mass, thus requiring an osteoanabolic agent instead of an anti-osteoporotic one, which provides the maximum gains with regard to BMD.

More in detail, teriparatide is a recombinant formulation of endogenous parathyroid hormone (PTH), and its pharmacological activity is similar to that of PTH. Therefore, it leads to a rapid increase in bone formation markers over bone resorption markers, creating the so called “anabolic window”, where the actions of PTH are maximally anabolic [6].

On the other hand, PEMF therapy is an instrumental physical therapy that uses electromagnetic fields to treat acute or chronic bone and muscle disorders, having a good therapeutic effect for pain relief [8]. It has an osteo-inductive effect, especially in conjunction with specific drugs. In this case report, it was used in combination with Teriparatide injection to reduce pain and inflammation and to promote healing of bone fractures, allowing earlier patient mobilization.

Several studies (Table 1) have so far reported the use of Teriparatide as effective treatment of bone fractures [[9], [10], [11]]. These include two retrospective case series that compare surgical approach with daily injection of Teriparatide for the treatment of intertrochanteric or thoracolumbar burst fractures, showing identical or even favorable results for those patients treated with Teriparatide. Moreover, a randomized controlled trial compares the combination of internal fixation surgery with either Teriparatide or placebo, showing no significant difference. However, only one of them [11] presents vertebral burst fractures, and compares the injection of Teriparatide only to vertebroplasty with Teriparatide injection, and to internal fixation surgery, showing no significant difference among the surgical and conservative treatments. Three case reports described vertebral fractures (T2, L2 and L1), similar to the one presented in this article [[12], [13], [14]]. In particular, 20 μg/day of Teriparatide for 6–12 months were administered, and in all cases the patients were free of pain and experienced bone callus formation and fracture healing after 6 months. The age of these patients were 56, 70 and 82-years. The patient presented in this article was 40 years old at the time of the trauma, thus younger than the56-year-old man.

**Table 1:** Articles reporting use of teriparatide for the treatment of bone fractures in patients with clear surgical indication.

Reference	Patients	Age	Fracture site	Treatment	Outcome
Bhandari et al. [9]	78	70	Femoral neck fracture	Internal fixation with teriparatide	No significant difference in terms of fracture healing
	81			Internal fixation with placebo	
Biro et al. [12]	1	56	T2	Teriparatide (20 μg/day for 6 months)	Complete fracture healing after 6 months
Kim et al. [10]	60	80.2	Intertrochanteric fracture	Internal fixation surgery	Better HHS, VAS and fracture healing in the group treated with teriparatide
	52	81.4		Internal fixation with teriparatide (for 2 months)	
Matsumoto et al. [13]	1	70	L2	Teriparatide	Bone formation at fracture level after 2 months
Park HY. et al. [14]	1	82	L1	Teriparatide (20 μg/day for 12 months)	Fracture healing after 6 months
Yu et al. [11]	12	73.77	Thoracolumbar fractures	Teriparatide (20 μg/day for 6 months)	No significant difference in terms of fracture healing
	12			Vertebroplasty with teriparatide (20 μg/day for 6 months)	
	11			Internal fixation	
Iwata et al. [15]	38	75.5	Osteoporotic vertebral burst fracture	Teriparatide (20 μg/day for 6 months)	Fracture union rate: 89 % at 6 months
	60	77.6		Bisphosphonates (35 mg/week for 6 months)	Fracture union rate: 68 % at 6 months
Park D. et al. [16]	23	73.3	Osteoporotic vertebral burst fracture	Teriparatide	No significant difference in terms of fracture healing
	32	73.8		Romosozunab	

Furthermore, our patient is not affected by osteoporosis nor other bone pathologies, while patients in the mentioned case reports were affected by other skeletal comorbidities as ankylosing spondylitis [12] and Diffuse Idiopathic Skeletal Hyperostosis [13]. To the best of our knowledge, there is no other case report of a patient suffering from vertebral bone burst fracture, without other bone pathologies, treated with the off-label therapy described previously.

Based on preliminary results of these previous studies, we decided to undergo off-label use of Teriparatide for a patient with L2 burst fracture, classified as an A2/A3 vertebral fracture and with a clear surgical indication.

Conservative treatment was preferred instead of surgery since the latter involves considerably higher risks associated to spinal cord injuries, and the former revealed optimal prognostic results for A type stable vertebral lumbar fractures. In addition, from an economic point of view, conservative pharmacological treatment is less expensive than surgical treatment, since it does not involve all the physical resources needed to perform this intervention. Moreover, the study from Yu et al., comparing surgical and conservative treatment, did not show a significant difference, in terms of functional and radiological outcomes, between drug injection therapy (Teriparatide) and surgical treatment [11]. Therefore, Teriparatide was used, and its osteogenic action was supported and empowered by the use of an external physical therapy (pulsed electro-magnetic field).

## Conclusion

This case report presents the off-label use of teriparatide and PEMF for the treatment of a L2 vertebral burst fracture in a young patient without any other bone pathologies or bone fractures. Other case reports in literature reported off-label use of teriparatide for the treatment of thoracolumbar vertebral compression fractures [[12], [13], [14]]. However, in these case reports, the patient was significantly older than 40 years old, and with other worsening pathologies. Therefore, this case report demonstrates the efficacy of Teriparatide as well as surgical internal fixation of vertebral fractures. Indeed, fracture healing was achieved avoiding the need for surgical intervention and the patient was free of symptoms after 5 months. Moreover, this is the only case in literature that uses electromagnetic field in addition to a conservative pharmacological therapy in a young patient without any other comorbidities.

## CRediT authorship contribution statement

**Tiziano Villa:** Writing – review & editing, Writing – original draft, Visualization, Validation, Supervision, Project administration, Methodology, Data curation, Conceptualization. **Vincenzo Zottola:** Writing – review & editing, Writing – original draft, Supervision, Conceptualization. **Carlo Mariani:** Writing – review & editing, Writing – original draft, Supervision, Conceptualization. **Alberto Borgonovo:** Writing – review & editing, Writing – original draft, Supervision. **Luciano Redenti:** Writing – review & editing, Validation.

## Funding

This research did not receive any specific grant from funding agencies in the public, commercial, or not-for-profit sectors.

## Declaration of competing interest

Authors declare no conflict of competing interest.
